# Oridonin induces growth inhibition and apoptosis in human gastric carcinoma cells by enhancement of p53 expression and function

**DOI:** 10.1590/1414-431X20187599

**Published:** 2018-11-14

**Authors:** Enxu Bi, Dengqiang Liu, Youxi Li, Xuying Mao, Aihua Wang, Jingtao Wang

**Affiliations:** 1 Department of General Surgery, Qingdao West Coast New Area Central Hospital, Qingdao, Shandong, China; 2 Department of Hepatopancreatobiliary Surgery, Huangdao Branch, The Affiliated Hospital of Qingdao University, Qingdao, Shandong, China

**Keywords:** Oridonin, p53, Gastric cancer, Cell apoptosis, Mdm2

## Abstract

The tumor suppressive role of oridonin, an active compound extracted from *Rabdosia rubescens*, has been proven in several gastric cancer (GC) cell lines. The present study aimed to evaluate the effect of oridonin on another GC cell line, SNU-216, and explore the potential mechanisms. The viable cell numbers, cell migration, survival fraction, and cell viability were, respectively, evaluated by trypan blue exclusion assay, wound healing assay, clonogenic assay, and CCK-8 assay. Cell apoptosis was determined by flow cytometry assay and western blot. The expression of p53 was inhibited by transient transfection, and the efficiency was verified by western blot. qRT-PCR was performed to measure the mRNA expression of p53. Western blot was used to evaluate the protein expression of apoptosis, DNA damage and p53 function related factors. We found that oridonin significantly inhibited cell proliferation, migration, and survivability, and enhanced cell apoptosis in SNU-216 cells. However, it had no influence on HEK293 cell viability. Oridonin also remarkably enhanced the anti-tumor effect of cisplatin on SNU-216 cells, as it significantly increased apoptotic cells and decreased cell viability. Moreover, the mRNA and protein expression of p53 was significantly up-regulated in oridonin-treated cells, while Mdm2 expression was down-regulated. Furthermore, oridonin enhanced p53 function and induced DNA damage. Knockdown of p53 or employing the caspase inhibitor, Boc-D-FMK, reversed the effect of oridonin on cell viability and apoptosis-related protein expression. The present study demonstrated that oridonin exhibited an anti-tumor effect on GC SNU-216 cells through regulating p53 expression and function.

## Introduction

Gastric cancer (GC) is the fourth most common cancer and the second most frequent cause of cancer-related deaths worldwide, particularly in East Asia, (1,2). Due to late-stage diagnosis and lack of sensitive biomarkers for early detection, the prognosis of GC is poor (3). Therefore, it is imperative to elucidate the regulatory network underlying GC and develop novel biomarkers or drugs for diagnosis and therapy.

Remarkable advances have been made in our understanding of cancer biology and cancer genetics. Among the most important of these advances is the realization that apoptosis and the genes involved in apoptosis have a profound effect on the malignant phenotype (4). One of the most effective methods for cancer therapy is the promotion of cell apoptosis by various cytotoxic anticancer agents (5). The transcriptional factor p53 is one of the most important tumor suppressors in cells, which promotes malignant cell death and maintains normal cell growth (6). It has been reported that several compounds exert the potent anti-tumor activity through targeting p53 and inducing cell apoptosis. For example, curcumin induces cell apoptosis in human breast cancer cells through a p53-dependent pathway in which Bax is the downstream effector of p53 (7). A small molecule, RITA, has been found to bind to p53, block p53-HDM-2 interaction, and enhance p53 function in tumors, thus suppressing their growth (8).

Oridonin is an effective diterpenoid isolated from *Rabdosia rubescens*, a herbal medicine that has been traditionally used in China for treating carcinoma of the digestive tract (9). It has been reported that oridonin exerts various pharmacological and physiological effects including anti-inflammation, anti-bacteria, and anti-tumor effects (10–12). Some reports have revealed that oridonin plays remarkable suppressive effects on breast carcinoma, non-small cell lung cancers, acute promyelocytic leukemia, and glioblastoma multiforme (13–15). For GC, the tumor suppressive role of oridonin has been reported in several cell lines, including MKN45 cells, HGC-27 cells, and SGC-7901 cells (16–18). It has been proven that oridonin can repress proliferation and elevate apoptosis of AGS cells, a GC cell line, via p53- and caspase-3-mediated mechanism (19).

Herein, we verified the effects of oridonin on proliferation, migration, apoptosis, and resistance to cisplatin on another gastric cancer cell line, SNU-216. The regulatory mechanism associated with p53 was also confirmed to enrich the experimental evidence for oridonin as a tumor suppressor in GC.

## Material and Methods

### Cell culture and treatment

The human GC cell line SNU-216 and human kidney epithelial cell line HEK293 were purchased from the American Type Culture Collection (ATCC, USA). Cells were cultured in Dulbecco's modified Eagle's medium (DMEM; Gibco, USA) containing 10% heat-inactivated fetal bovine serum (FBS; Gibco). The cells were seeded onto culture dishes at 37°C in a humidified 5% CO_2_ incubator. Oridonin (Sigma-Aldrich, USA) was dissolved in DMSO and diluted into adequate volume of DMEM.

### siRNA transfection

Small interfering RNAs (siRNAs) against p53 (si-p53 #1 and si-p53 #2) and their negative control (NC) were purchased from Cell Signaling Technology (USA). siRNAs were respectively transfected into SNU-216 cells in 96-well plates or 6-well plates using Lipofectamine 2000 reagent (Life Technologies Corporation, USA) on the basis of the manufacturer's instructions. At 48 h post-transfection, the efficiency of gene silencing was measured via western blot.

### Trypan blue dye

SNU-216 cells were plated in 24-well plates at a concentration of 1×10^5^ cells/well. When the cells reached attachment, cells were exposed to a series of doses of oridonin (0, 10, 40, 80 μM) and incubated for 0-5 days. Cells were trypsinized and stained with trypan blue dye (Beyotime Biotechnology, China), and viable cells were counted using cell counting chamber every 24 h for a total of 5 days. Viable cell numbers of each group were collected and used to plot the cell growth curves.

### Cell viability assay

Cell viability was determined by the Cell Counting Kit-8 (CCK-8; Dojindo Molecular Technologies, USA) according to the manufacturer's instructions. In brief, 5×10^3^ SNU-216 cells were seeded per well in 96-well plates. After treatment with oridonin, 10 μL of CCK-8 solution was added to the culture medium and cells were incubated for another 1 h in humidified 95% air and 5% CO_2_ at 37°C. Then, the absorbance was measured at 450 nm on a microplate reader (Bio-Rad, USA).

### Wound healing assay

SNU-216 cells were plated in 60-mm dishes until 80–90% confluence and were treated with 50 μM mytomicin C (Sigma-Aldrich) for 3 h. Wounds were then created by scratching cell sheets with a sterile 200-μL pipette tip and cells were treated with oridonin. Cell migration was monitored according to the images taken by an inverted microscope (Leica DMIRB, Leica Microsystems, Germany) every 24 h. The distance between one side of the scratch and the other were detected and the relative wound widths were calculated. Data are reported as means±SD of 3 independent experiments.

### Clonogenic assay

For the clonogenic assay, 1×10^3^ cells were plated in 60-mm dishes and cultured in medium containing DMSO or oridonin for 2 to 3 weeks. Then, cell clones were rinsed with PBS, fixed with ice methanol and stained with 10% Giemsa (Sigma-Aldrich). Images of stained cell clones on plates with different treatments were captured, and the surviving fraction was calculated as a ratio of the number of treated cells to the number of cells plated (plating efficiency) divided by the same ratio calculated for the non-treated group.

### Apoptosis assay

The annexin V-FITC/PI apoptosis detection kit (Beijing Biosea Biotechnology, China) was used to identify and quantify the apoptotic cells. Treated cells were harvested and washed with PBS, and then resuspended in 1 × binding buffer. Cells were double-stained with annexin V-FITC (5 μg) and PI (5 μg) according to the manufacturer's protocol. Apoptotic cells were measured and analyzed on a FACScan (Beckman Coulter, USA) and the data were analyzed using FlowJo software (Tree Star Inc., USA).

### Quantitative reverse transcription PCR (qRT-PCR)

Total RNA was extracted with TRIzol reagent (Life Technologies Corporation) following the manufacturer's instructions. Total RNA (500 ng) was reversely transcribed to cDNA using the PrimeScript RT reagent kit (TaKaRa, China). Real-time PCR was performed on the Bio-Rad CFX 96 Real-time PCR system using SYBR® Premix Ex TaqTM II (TaKaRa). The mRNA level of p53 was normalized to β-actin with the 2^-ΔΔCt^ method (20).

### Western blot

The protein used for western blot analysis was extracted using RIPA lysis buffer (Beyotime Biotechnology) containing protease inhibitors (Roche, Switzerland). Equivalent proteins (30 μg) were loaded on the sodium dodecyl sulfate-polyacrylamide gel electrophoresis (SDS-PAGE) and transferred onto the polyvinylidene difluoride (PVDF) membranes (Millipore, USA). The membranes were blocked with 5% bovine serum albumin (BSA; Roche) for 2 h at room temperature, followed by incubation with primary antibodies overnight at 4°C. The primary antibodies were prepared in 5% BSA at a dilution of 1:1000. Primary antibodies used in the study were as follows: anti-Bcl-2 (#4223), anti-Bax (#5023), anti-caspase-3 (#9662), anti-caspase-9 (#9502), anti-PARP (#9532), anti-p53 (#2524), anti-p-p53 (#9284), anti-p21 (#2947), anti-Mdm2 (#86934), anti-γ-H2AX (#9718), and anti-β-actin (#4970; Cell Signaling Technology). Then, blots were rinsed with Tris-buffered saline plus Tween-20 (TBST) and incubated with secondary antibody marked by horseradish peroxidase for 1 h at room temperature. After being rinsed, the membranes were subjected to the ChemiDocTM XRS system (Bio-Rad), and Immobilon Western Chemiluminescent HRP Substrate (Millipore) was added. The signal intensity of each band was captured and quantified using Image Lab^TM^ software (Bio-Rad).

### Statistical analysis

All experiments were repeated three times. The results of multiple experiments are reported as means±SD. Statistical analyses were performed using Graphpad Prism 6 software (GraphPad Software, USA). P values were calculated using one-way analysis of variance (ANOVA). A P value <0.05 was considered to indicate a statistically significant result.

## Results

### Oridonin inhibited the growth, migration, and survivability of SNU-216 cells

**Figure 1.**
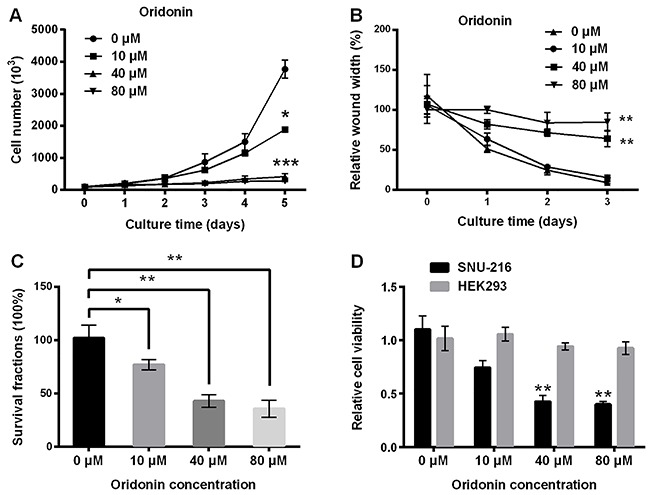
Oridonin inhibited the growth, migration, and survivability of SNU-216 cells. Cells were treated with a series of oridonin (0, 10, 40 and 80 μM) for indicated days. *A*, Viable cell number was measured by trypan blue dye staining. *B*, Cell migration was assessed by wound healing assay. After treatment with oridonin for 24 h, the survival fraction of cells was detected by clonogenic assay (*C*), and cell viability of SNU-216 cells and HEK293 cells was measured by CCK-8 assay (*D*). Data are reported as means±SD. *P<0.05, **P<0.01, ***P<0.001 compared to 0 μM (ANOVA).

As shown in Figure 1A, the number of cells in the control group (non-treated cells) was increased in a time-dependent manner. However, after treatment of 10, 40, and 80 μM oridonin, cell numbers were significantly decreased compared with the control group (P<0.05 or P<0.001). Wound healing analysis revealed that 40 and 80 μM oridonin significantly suppressed cell migration (Figure 1B) (both P<0.01). Results in Figure 1C show that oridonin significantly reduced the survival fraction of SNU-216 cells in a dose-dependent manner (P<0.05 or P<0.01). Furthermore, we investigated whether the effect of oridonin on SNU-216 cells was selective. The HEK293 cells and SNU-216 cells were both treated with oridonin and cell viability was measured. As shown in Figure 1D, 40 and 80 μM oridonin significantly reduced the cell viability of SNU-216 cells compared to non-treated cells (both P<0.01), while it had no influence on HEK293 cells (P>0.05). These results suggested that oridonin selectively inhibited the growth, migration, and survivability of SNU-216 cells.

### Oridonin induced apoptosis in SNU-216 cells

**Figure 2.**
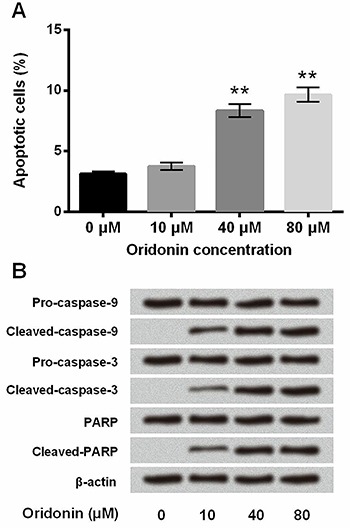
Oridonin promoted apoptosis in SNU-216 cells. SNU-216 cells were treated with oridonin (0, 10, 40 and 80 μM) for 24 h. *A*, Percentage of apoptotic cells was determined by flow cytometry assay, and *B*, expression of apoptosis-related proteins was detected by western blot. Data are reported as means±SD. **P<0.01 compared to 0 μM (ANOVA).

Flow cytometry analysis revealed that the apoptotic cell rate of SNU-216 cells was notably increased by oridonin (40 and 80 μM) compared to the non-treated cells (Figure 2A) (both P<0.01). Similar results were observed in western blot analysis; oridonin increased the expression of pro-apoptotic proteins (cleaved caspase-3, cleaved caspase-9, and cleaved PARP) (Figure 2B). These results indicated that oridonin promoted cell apoptosis in SNU-216 cells.

### Oridonin synergized the effect of cisplatin in SNU-216 cells

**Figure 3.**
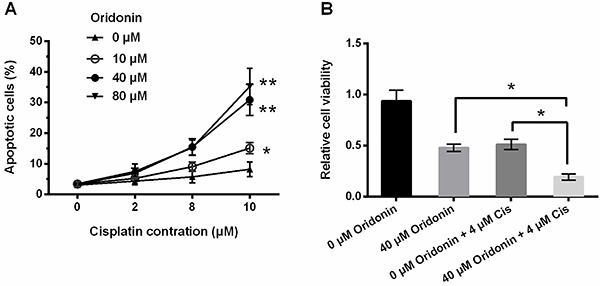
Oridonin synergized the effect of cisplatin in SNU-216 cells. SNU-216 cells were treated with oridonin combining with or without cisplatin for 24 h. *A*, percentage of apoptotic cells and *B*, cell viability were assessed by flow cytometry assay and CCK-8 assay, respectively. Cis, cisplatin. Data are reported as means±SD. *P<0.05, **P<0.01 (ANOVA).

Since cisplatin is an important chemotherapeutic drug in the treatment of GC, we further investigated whether it could function as a synergist of cisplatin in GC cells. SNU-216 cells were treated with oridonin combining with or without cisplatin, and subjected to flow cytometry and cell viability assays. Results in Figure 3A show that the combination of oridonin and cisplatin induced a more pronounced increase of apoptotic cell rates (P<0.05 or P<0.01). As shown in Figure 3B, the combination of oridonin (40 μM) and cisplatin (4 μM) significantly decreased cell viability of SNU-216 cells compared with the treatment of oridonin (40 μM) or cisplatin (4 μM) alone (both P<0.05). Overall, these results suggested that oridonin synergized the anti-tumor effect of cisplatin in SNU-216 cells.

### Oridonin enhanced p53 expression by inhibition of Mdm2 in SNU-216 cells

**Figure 4.**
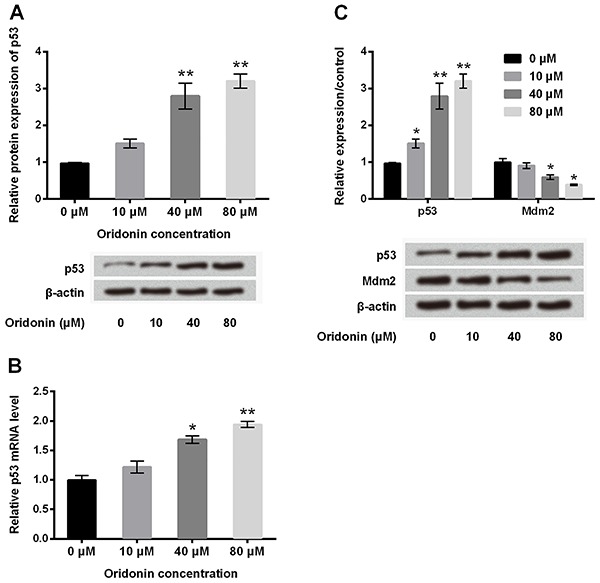
Oridonin enhanced p53 expression by inhibition of Mdm2 in SNU-216 cells. SNU-216 cells were exposed to oridonin (0, 10, 40, and 80 μM) for 24 h. The protein (*A*) and mRNA (*B*) expressions of p53, and the protein expressions of p53 and Mdm2 (*C*) were determined by western blot. Data are reported as means±SD. *P<0.05, **P<0.01 compared to 0 μM (ANOVA).

We then investigated the underlying mechanism of oridonin in SNU-216 cells. Transcriptional factor p53, an important tumor suppressor, can protect normal cell growth and initiate malignant cell death (6). We examined the effect of oridonin on p53 expression. As shown in Figure 4A and B, protein and mRNA expressions of p53 was up-regulated in oridonin-treated cells in a dose-dependent manner (P<0.05 or P<0.01). It has been reported that the level and activity of p53 are strictly modulated by the ubiquitin E3 ligase Mdm2, which binds p53 directly and mediates p53 ubiquitination and proteasomal degradation continuously (21). We further explored the effect of oridonin on Mdm2 expression. Results in Figure 4C show that oridonin significantly up-regulated the expression of p53, and down-regulated the expression of Mdm2 (P<0.05 or P<0.01). These results suggested that oridonin might up-regulate p53 expression, possibly by inhibition of Mdm2, in SNU-216 cells.

### Oridonin enhanced the functions of p53 in SNU-216 cells

**Figure 5.**
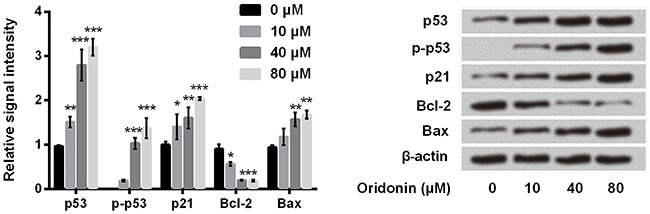
Oridonin enhanced the functions of p53 in SNU-216 cells. SNU-216 cells were incubated with oridonin (0, 10, 40, and 80 μM) for 24 h. Then, the protein levels of p53, p-p53, p21, Bcl-2, and Bax were detected by western blot. Data are reported as means±SD. *P<0.05, **P<0.01, ***P<0.001 compared to 0 μM (ANOVA).

p53 induces cell cycle arrest, DNA damage repair and initiation of apoptosis often through binding to the response element on downstream target genes and activates or represses expression of these genes (21). To investigate the effect of oridonin on the functions of p53, we assessed the expression of several downstream factors of p53 by western blot, such as p21, Bcl-2, and Bax, and detected the phosphorylation of p53. We found that oridonin significantly promoted the expression of p53, p-p53, p21, and Bax, and inhibited the expression of Bcl-2 (Figure 5) (P<0.05, P<0.01, or P<0.001). These results demonstrated that oridonin not only promoted p53 expression but also enhanced p53 function.

### Oridonin induced DNA damage in SNU-216 cells

**Figure 6.**
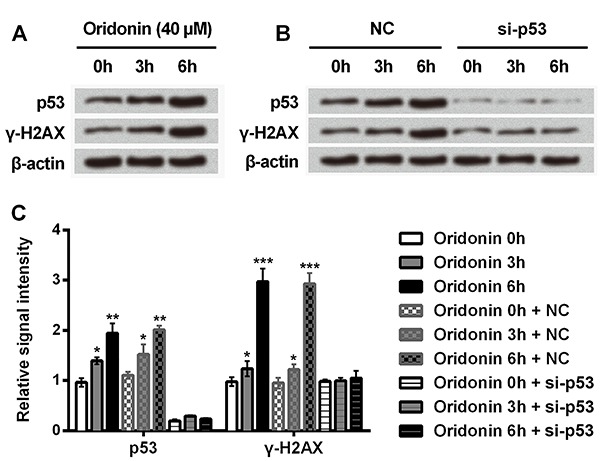
Oridonin induced DNA damage in SNU-216 cells. SNU-216 cells were transfected with si-p53 to silence p53 expression. Then, transfected cells or non-transfected cells were treated with oridonin (40 μM) for 0 h, 3 h, and 6 h. The protein expression of p53 and γ-H2AX was measured by western blot. *A*, Blots of p53 and γ-H2AX in non-transfected cells. *B*, Blots of p53 and γ-H2AX in transfected cells. *C*, Quantification of relative signal intensity of blots in non-transfected cells and transfected cells. NC, negative control of si-p53. Data are reported as means±SD. *P<0.05, **P<0.01, ***P<0.001 compared to 0 h (ANOVA).

As we have demonstrated that oridonin enhanced p53 expression and function, we hypothesized that oridonin might induce DNA damage in SNU-216 cells. γ-H2AX (phosphorylated histone H2AX on serine 139) is a sensitive DNA damage marker specifically induced by DNA double-strand breaks (DSB) (22). Thus, we assessed the effect of oridonin on the expression of γ-H2AX in SNU-216 cells. As shown in Figure 6A and C, treatment of 40 μM oridonin for 3 h and 6 h significantly increased expression of p53 and γ-H2AX relative to non-treated cells (P<0.05 or P<0.01). Furthermore, SNU-216 cells were transfected with si-p53 to inhibit p53 expression, and we then investigated the effect of oridonin on p53 and γ-H2AX expression. As shown in Figure 6B and C, oridonin had no influence on the expression of p53 and γ-H2AX in si-p53-transfected cells. These results revealed that p53 was involved in the DNA damages induced by oridonin.

### Role of p53 and caspase in the anti-tumor effect of oridonin

**Figure 7.**
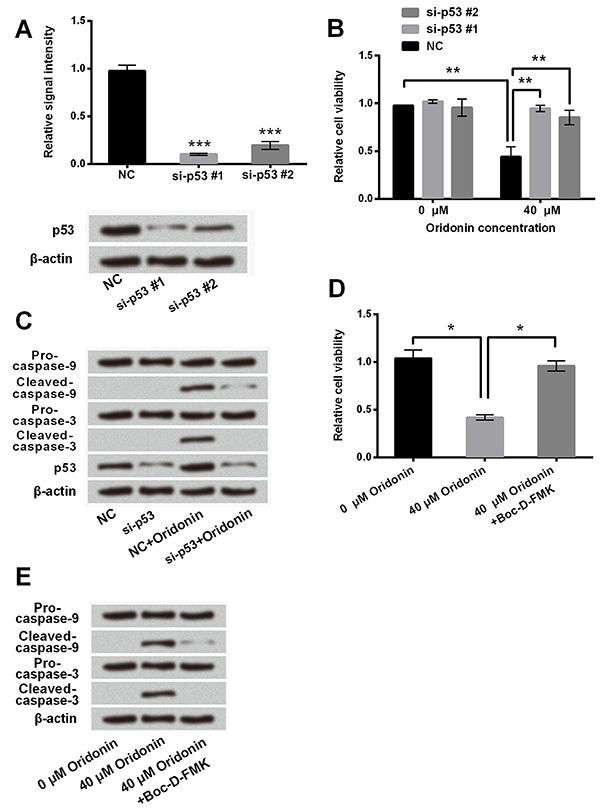
Role of p53 and caspase in the anti-tumor effect of oridonin. SNU-216 cells were transfected with si-p53 #1 or si-p53 #2. *A*, Transfected efficiency was verified by western blot. SNU-216 cells were transfected with si-p53 #1 or si-p53 #2, and then the transfected cells were exposed to oridonin (40 μM) for 24 h. *B*, Cell viability was measured by CCK-8 assay. *C*, Expression of p53, cleaved-caspase-3, and cleaved-caspase-9 was determined by western blot. SNU-216 cells were incubated with oridonin (40 μM) or combined with caspase inhibitor, Boc-D-FMK. *D*, Cell viability and *E*, expression of cleaved-caspase-3 and cleaved-caspase-9 were measured by CCK-8 assay and western blot, respectively. NC, negative control of si-p53 #1 and si-p53 #2. Data are reported as means±SD. *P<0.05, **P<0.01, ***P<0.001 (ANOVA).

To explore the relationship between oridonin-induced up-regulation of p53 and cell growth inhibition in SNU-216 cells, we assessed the effect of p53 inhibition on the anti-tumor activity of oridonin. As expected, the expression of p53 was significantly down-regulated in si-p53 #1-transfected and si-p53 #2-transfected cells (Figure 7A) (P<0.001). As shown in Figure 7B, oridonin (40 μM) markedly reduced the cell viability in NC-transfected cells (P<0.01). However, p53 knockdown significantly increased cell viability in cells under oridonin treatment, especially si-p53 #1 (Figure 7B) (both P<0.01). Thus, si-p53 #1 was used in subsequent western blot analysis. As shown in Figure 7C, knockdown of p53 by siRNA blocked the regulatory effect of oridonin on expression of apoptosis-related factors, as observable by the down-regulation of cleaved-caspase-9 and cleaved-caspase-3. Furthermore, we detected the effect of caspase inhibitor on oridonin cytotoxicity. We found that the caspase inhibitor Boc-D-FMK reversed the effect of oridonin on cell viability and expression of apoptosis-related proteins, confirmed by obvious enhancement of cell viability (Figure 7D) (P<0.05) and down-regulation of cleaved-caspase-9 and cleaved-caspase-3 (Figure 7E). Taken together, these results demonstrated that the anti-tumor effect of oridonin on SNU-216 cells might be associated with inhibition of cell apoptosis.

## Discussion

Oridonin has been reported to have tolerable toxicity and possess an inhibitory effect on a variety of human malignancies, such as cervical cancer (23), pancreatic cancer (2), hepatocellular carcinoma (24), fibrosarcoma (25), and others (26). In the present study, we demonstrated that oridonin possessed an anti-tumor effect in GC SNU-216 cells, as it inhibited cell proliferation and migration, and promoted cell apoptosis. These results were consistent with a previous study showing that oridonin exhibited tumor suppressive effects on gastric cancer cells (19).

Cisplatin is a first-generation platinum-based chemotherapeutic agent broadly used in the treatment of cancer. It has been reported that oridonin could reverse the cisplatin drug resistance in human ovarian cancer cells and mouse sarcoma S180 cells (27,28). We also investigated the effect of oridonin on resistance of SNU-216 cells to cisplatin. Our results showed that oridonin synergized the effect of cisplatin on cell viability and apoptosis in SNU-216 cells, which was consistent with previous reports. However, the potential mechanisms need to be further explored.

p53 acts as a crucial tumor suppressor in cells, the expression of which can be enhanced by cytotoxic stresses and lead to cell cycle arrest and apoptosis (21,29). The expression of p53 is regulated by various mechanisms, such as transcription, translation, ubiquitination-mediated degradation, mRNA and protein stability (21,29). Previous studies have revealed that oridonin inhibited proliferation of a wide variety of cancer cells through regulation of p53. For example, when p53 was suppressed by overexpression of E6 from human papilloma virus type 16 (HPV-16), prostate cells (LNCaP), and non-small cell lung cancer cells (NCI-H520) lost their sensitivity to oridonin-induced growth inhibition and apoptosis (15). In liver cancer cells (HepG2), oridonin has been found to induce G2/M cell cycle arrest and apoptosis through the MAPK and p53 signaling pathways (30). In pancreatic cancer, oridonin treatment significantly induced apoptotic cell death in SW1990 cells, and the regulatory mechanisms might be through p53- and caspase-dependent activation of p38 MAPK (31). Thus, we hypothesized that p53 expression and function might be associated with the suppressive effect of oridonin on cell growth and migration in SNU-216 cells. In the current study, we found that oridonin significantly increased the mRNA and protein expression of p53 in a dose-dependent manner. Furthermore, oridonin reduced the protein expression of Mdm2. Mdm2, widely proven to be a negative regulator of p53, represses p53 transcriptional activity and mediates the degradation of p53 (32,33). Our results implied that oridonin might increase p53 expression by down-regulating Mdm2 expression.

The important transcription factor p53 has multiple functions (34). It could bind to the response element on down-stream target genes and activate or repress expression of these genes, leading to cell cycle arrest, DNA damage repair, and initiation of apoptosis (21). Furthermore, p53 also could directly link with many cell pathways to induce DNA repair or apoptosis (29). We thus measured the expression of p53 target genes and apoptosis-related proteins that are regulated by p53 to investigate the effect of oridonin on p53 function. We found that oridonin enhanced p53 function, as it promoted the expression of p21, p-p53, and Bax, and inhibited Bcl-2 expression. Then, we further investigated whether oridonin could induce DNA damage via p53. We altered p53 expression by transfection with si-p53 and examined the expression of γ-H2AX, a sensitive DNA damage marker. We found that oridonin increased γ-H2AX expression, whereas the increase was blocked by p53 knockdown.

Oridonin has been reported to induce cell apoptosis and cell cycle arrest in many cancers, and our results revealed that oridonin inhibited cell growth, promoted cell apoptosis, and enhanced p53 expression and function. To investigate the relationship between oridonin-induced up-regulation of p53 and cell toxicity in SNU-216 cells, we analyzed the effect of p53 knockdown on the anti-inhibitory effect of oridonin. We found that p53 knockdown reversed the suppressive effect of oridonin on cell growth. We also found that the caspase inhibitor, Boc-D-FMK, blocked the effect of oridonin on cell viability as well as activation of caspase-3 and caspase-9. These results implied that apoptosis induction might be the main action mode by which oridonin elicits its anti-tumor effect in SNU-216 cells.

In conclusion, we revealed the anti-tumor effect of oridonin on SNU-216 cells, evidenced by inhibition of cell viability, migration, survivability, and increase of apoptosis. The regulatory mechanism of oridonin in SNU-216 cells might be enhancement of p53 expression and function. Our results may enrich experimental evidence for the anticarcinogenic role of oridonin in GC, providing a theoretical basis for its application as a potential drug for GC.
